# Improving adolescent health literacy through school-based health literacy intervention: a mixed-method study protocol

**DOI:** 10.1186/s12889-023-15316-4

**Published:** 2023-02-28

**Authors:** Shanti Prasad Khanal, Chitra Bahadur Budhathoki, Orkan Okan

**Affiliations:** 1 Central Department of Education, Faculty of Education, Tribhuvan University, Kirtipur, Nepal; 2 TUM Department of Sport and Health Sciences, Technical University , Munich, Germany

**Keywords:** Health literacy, Adolescent students, Health-promoting actions, Intervention

## Abstract

**Background:**

Health-promoting actions might benefit from adolescent health literacy (AHL), however, there is scant research on it in Nepal. This study identifies adolescent students’ health literacy (HL) needs and trials an intervention to improve their HL and intention to take health-promoting actions.

**Methods:**

This study employs a pre-and post-test mixed-method intervention involving three phases. First, we will conduct a formative and summative evaluation to identify participants’ HL needs and design an intervention using quantitative and qualitative methods. Second, the intervention will be administered to the intervention group. Finally, formative and summative post-tests will be conducted to assess the effectiveness of the intervention. We will select four community schools from Birendranagar municipality based on random sampling. In quantitative research, data will be collected from adolescents selected through a census with standardized scales such as the HLS-Child-Q15, self-efficacy, social support, and health-promoting actions. A framework analysis was conducted to analyze qualitative data collected from focus group discussions with purposively chosen adolescents and key informant interviews with Health and Physical Education teachers and school nurses. The *difference in difference* approach will be used to analyze the intervention’s outcome, i.e., the participants’ improved HL, and health-promoting actions.

**Discussion:**

This is one of the first studies to explore HL in this group in Nepal. This study will provide the first insights into the overall level of AHL, potential AHL determinants, and the relationship between AHL and the intention to participate in health-promoting activities. The data can then be used to inform health promotion and health literacy initiatives.

## Background

HL is a relatively new research field and an essential part of public health [1–4] and health promotion [5, 6]. Schools can play an influential role in promoting HL [7] by delivering health information through mandatory courses on health education to all school-aged children [8]. This makes HL a viable target for schools. Health education and promotion make a significant contribution to HL [5] and could be considered as a part of the Health Promoting School (HPS) [9, 10]. The growing interest in adolescent health literacy  (AHL) in recent years has been discussed worldwide [9]. Moreover, it has been argued that AHL research is crucial [11], but received far less attention than research on adults’ HL in many contexts [9].

The understanding of AHL is limited due to a paucity of studies [9, 12, 13], particularly in developing countries [14]. Evidence shows that low AHL is common even in developed nations [15–17]. For example, the majority of school-going students (67.2%) in ten European countries have only a moderate level of HL [18, 19]. Studies show that the HL of school-aged children is low. According to a study on Thai school-aged children, 64.4% reported poor levels of HL [20], followed by one-third for Chinese children [21]; 20.9% of Pakistani children had very limited, and 53.4% limited HL [22] and almost (99.3%) of Iranian students from 15 to 18 had poor HL [23]. Only a few HL studies have been conducted in Nepal, revealing that the majority of female college students [24, 25] and school adolescents have inadequate HL scores [26]. These studies provide the first insights into AHL, but the overall distribution of AHL remains unclear [21] and differs among countries and subgroups [18]. As per our current understanding, not much is known about the distribution of AHL in the population [27].

The next issue is that there is currently a scarcity of theory-driven HL interventions in schools to increase AHL. A limited number of studies have been conducted to develop interventions to promote generic HL in adolescents. For example, a recent review found that no intervention has explicitly aimed to improve HL [28] and the majority of existing school-based HL interventions are domain-specific without a holistic model [29]. It is frequently ignored in school curricula [30] and an effective strategy for improving HL in schools is still lacking [31]. Further, the literature shows that most of the studies conducted on the HL of adolescents are cross-sectional designs. To our knowledge, this will be the first intervention study to promote health literacy among adolescent school students in Nepal. This is why a longitudinal research design is employed in this study.

## Aims

Based on the available evidence, this study aims to.

(i) explore the HL needs of adolescent students and.
(ii) design, implement and evaluate the impact of a health literacy educational intervention to improve adolescent students’ health literacy and their intention to take health-promoting actions.

## Methods

### Research method

The study follows a pre-and post-test [32, 33], mixed-method intervention design [34] grounded in pragmatism. Researchers have identified pragmatic research as the preferred approach to mixed-methods research [32, 33, 35, 36].

### Theoretical model

**Fig. 1:**
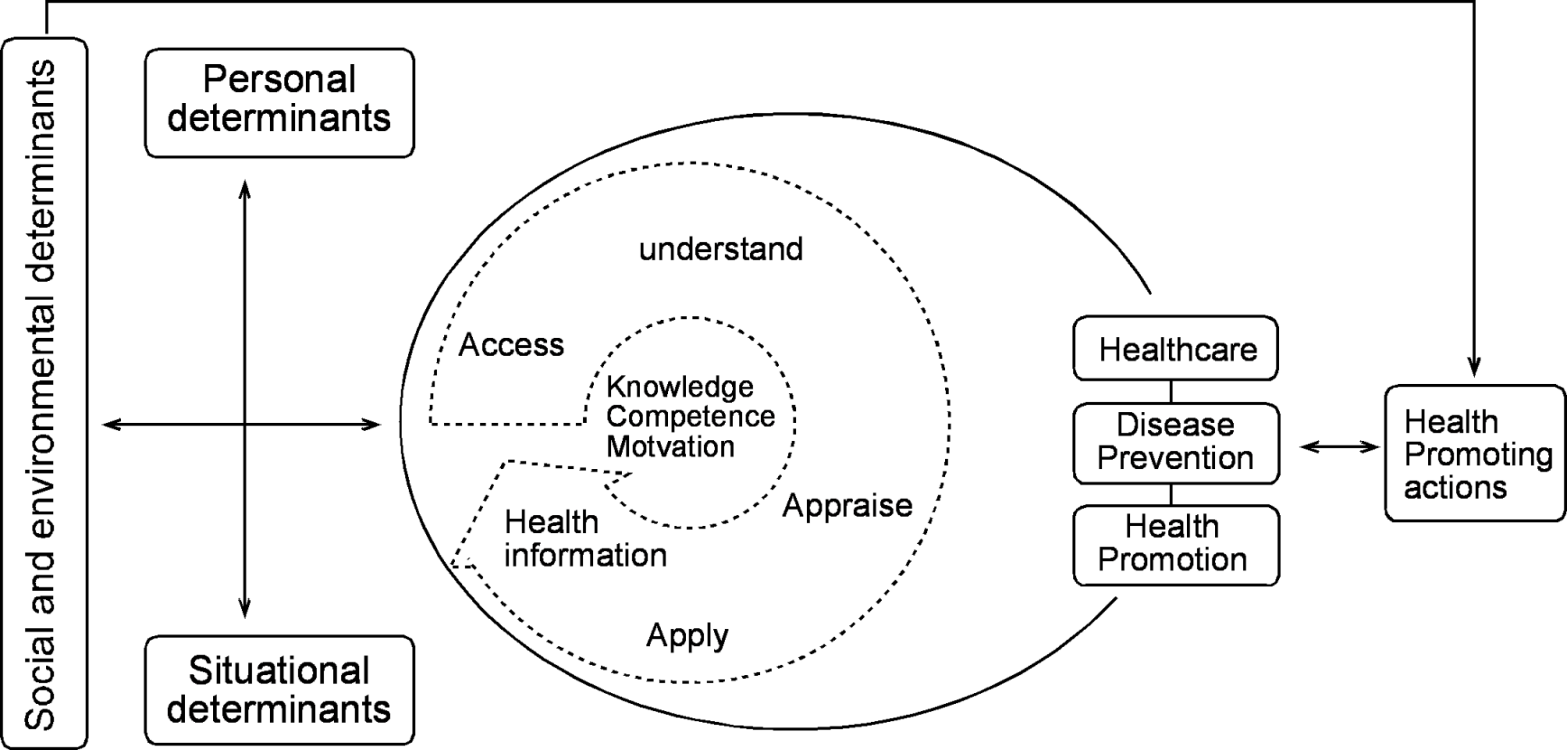
Simplified HLS-EU model of health literacy [40] adapted to the study design and needs

While reviewing the various models [37–39] in light of this study’s overall goals, the comprehensive and conceptual model appears to be applicable as a theoretical model [40]. The understanding of HL within this study is underpinned by the four core dimensions of the HL Model as presented by Sorensen et al. (2012): (1) accessing, (2) understanding, (3) appraising, and (4) applying health information. Based on this model, this study will explore the multiple determinants that shape HL (Fig. 1), namely (1) personal, (2) situational, as well as (3) societal and environmental determinants. All three factors influence how adolescents deal with health information. Also, the model covers many potential outcomes of HL. Of these outcomes, the study will cover only health-promoting actions as a specific form of health behaviors [6].

### Study site

This study will be conducted in the governmental school of Birendranagar Municipality of Surkhet. There are a total of 21 schools in the municipality where 2500 (average: 156 per school) students are studying in class nine in this academic session [41]. Karnali Province is less developed, has low human development indicators, and has a low literacy rate compared to other provinces of Nepal [42, 43]. Birendranagar is an educational and cultural hub where students from different districts of Karnali province come to study. Therefore, it has been selected as a study area.

### Research design

**Fig. 2:**
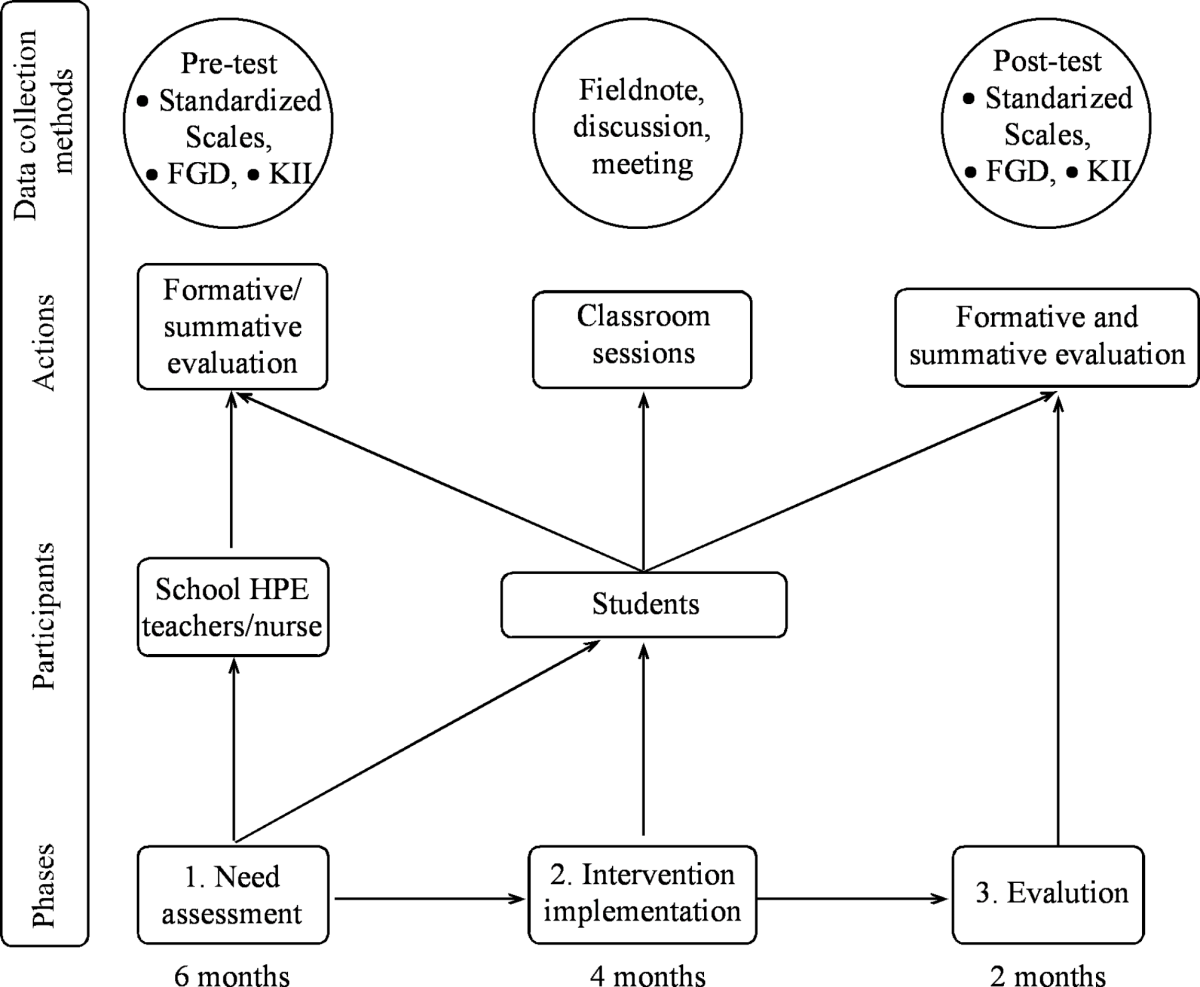
Overview of the research design

This study contains three phases (see Fig. 2). Formative and summative evaluation will be used to explore the impact of the intervention. Formative assessment will assess the feasibility and acceptability of interventions and help inform their implementation [34]. Summative evaluation will be used to investigate the impact of the intervention.

### Phase 1 needs assessment

As part of the formative evaluation [34], all class nine students, as well as their Health and Physical Education (HPE) teachers and school nurses from the four schools will take part in quantitative and qualitative pre-tests. This phase will assess participants’ HL knowledge, and, needs. This phase [44] will also inform the implementation strategies [34, 45].

**Fig. 3:**
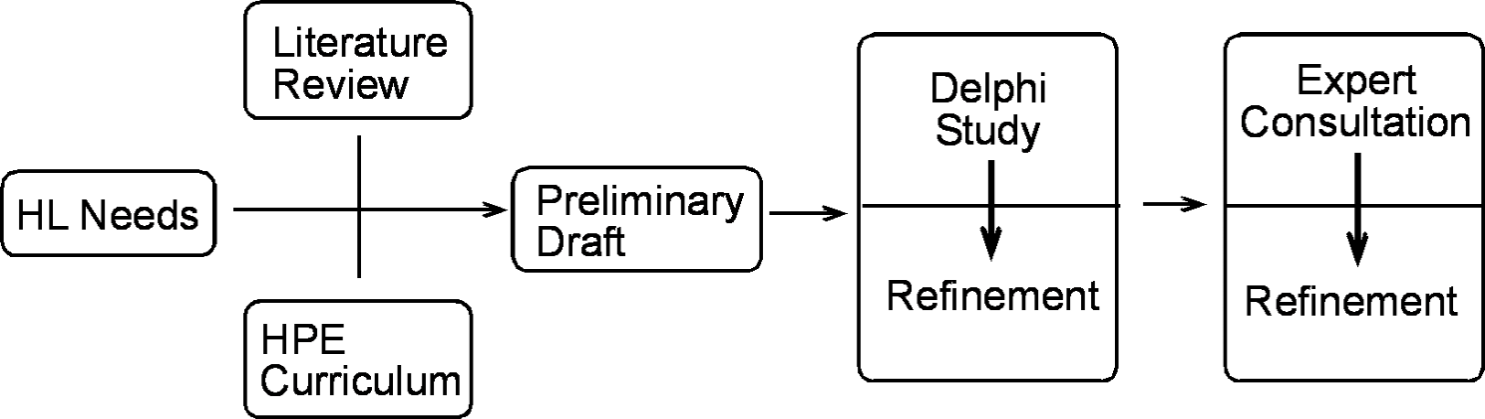
Flow chart of the intervention development process

The HL Intervention will be developed in three steps. First, the researchers will prepare a preliminary draft of the intervention based on the IM’s four dimensions (accessing, understanding, appraising, and applying health information [40]), and the HPE curriculum of class nine. Second, the preliminary draft will be refined based on the HL needs of the target group. This will then be further refined by the Delphi study with HPE teachers, school nurse, health education, public health and medical care experts. As part of this process, the 9th -grade HPE curriculum will be integrated into the interventions’ contents, strategies, materials, and practical facilitation of the sessions. Third, to make it more aligned with HL needs, the curriculum, and the HL domains (accessing, understanding, evaluating, and applying), the refined draft will be finalized based on consultation with health educators and health literacy experts (see Fig. 3).

### Phase 2 implementation

In this phase, the refined intervention will be implemented in the intervention group in eight separate classroom sessions. One session will last for 1 h to 90 min. Teachers from participating schools, HPE teachers from participating local campuses, public health officers, and medical officers will conduct the sessions (Table 1).

**Table 1:** Preliminary intervention draft based on IMHL [40] and HPE curriculum

Input	Sessions	Objectives	Contents	Activities	Outcome
Program introduction	Researcher	To become familiar with the program	Introduction to program, and HL and health-promoting actions	Group teaching and interaction	Increased HL awareness and skills Intention to engage in health promotion actions
Human resources Time Money Materials Technology Partnership	Health education experts	to raise adolescents’ awareness of health literacy.	Health Literacy (HL) concept	Storytelling and Dialogue	
	HPE teacher/ school nurse	To develop Confidence in health literacy skills	Health information: access, understand, appraisal, and apply	Clear communication, photograph showing demonstration, experience sharing	
	Teacher’s educator	Confident to evaluate the determinants of health	Social determinants of health, health right, and responsibility	Video showing and Dialogue	
	Medical doctors	Confident to use health care	Health care literacy	Clear communication, role playing	
	School Nurse	To raise awareness on disease prevention	Disease prevention literacy	Experience sharing, playing game	
	Public health experts	To raise awareness on disease prevention	Health promotion literacy	self-assessment and reflection of text and presentation	
	Health education experts	to pursue health-promoting activities	Health-promoting actions	Case study, video show, playing game, group discussion	

### Phase 3 evaluation

Both formative and summative evaluation methods will be employed to evaluate the impact of the health literacy educational intervention. Based on the above description, pre-interventional evaluation [34] information will be collected from students, HPE teachers, and nurses through quantitative and qualitative pre-tests, as well as a review of the grade nine curriculum. Discussion and meetings will be conducted with HPE teachers, students, and facilitators during the implementation process. This is to determine whether the plan is being implemented properly and whether there are any discrepancies [46].

To evaluate the overall success, usefulness, satisfaction, and additional suggestions [34], contextual appropriateness, relevancy, and acceptability [44] of the intervention, interpretative formative evaluation [46] will be used. Summative evaluation will be used to assess the overall effectiveness of the intervention at the end of the project.

### Participants and sampling

The study will involve adolescent students in grade nine as primary participants, and HPE teachers and school nurses as secondary participants. Since academic grades and age affect HL, only grade nine will be taken for this study to construct the same group of intervention and control [39].

#### Sampling for a quantitative study

Firstly, four governmental secondary schools will be chosen from the study area using a random sampling method for the needs assessment. Then, two of the four selected schools will be considered as an intervention group randomly and the remaining two schools will be considered as a control group. The sample size of this study will be at least 384. All students of class nine of the selected schools will be selected via the census. Assuming that the margin of error is 5% and the level of confidence is 95%, the recommended standard sample size (SSS) is 384 [47]. Non-response samples will be added after the pilot test.

The formula of SSS [47]: $n={\text{z}}^{2}\left(p\right)\left(q\right)/\left(e\right)$[where n= sample size, Z= standard error associated with the chosen level of confidence, p= variability/, q = 1-p, and e= acceptable sample error] will be used.

#### Sampling for a qualitative study

Twenty-four (six from each school) adolescents will be purposefully selected from the participating schools. Similarly, HPE teachers and school nurses of the respective schools will also be included as key informants for the study.

### Inclusion and exclusion criteria

All students in class nine of selected schools in Surkhet, aged 13 to 19, who are willing to participate in the study, will be included. However, students with special needs and who skip two educational sessions will be excluded from the intervention.

### Tools and materials

#### Quantitative data collection tools

Information about participants’ characteristics such as age, sex, caste, religion, parental education, family size, availability of TV, radio, mobile, internet, health status, educational outcome, and HL knowledge will be collected using a self-administered questionnaire. To collect quantitative data relating to the pre-test and post-test, standardized tools will be used, such as self-efficacy, social support, school environment, community environment, HLS CHILD-15, and health-promoting action scale (Table 2).

**Table 2:** *Standardized data collection tools*

SN	Variable	Tools	Literature	Total items and components	Reliability	Validity
1	Personal factors	General Self-efficacy Scale (GSE)	R Schwarzer and M Jerusalem [48]	10 items 4 scales, ranges between 10 and 40	α = 76 to 0.90	Strong structural validity
2	Situational factors:	Multi-dimensional scale for perceived social support Scale (MSPSS)	GD Zimet, NW Dahlem, SG Zimet and GK Farley [49]	12-item scale family (4), friends (4), significant other (4).	α = 0.86–0.93)	good construct validity
3	School Environment	The school environment scale (SES)	R Glaser, ML Van Horn, M Arthur, J Hawkins and R Catalano [50]	17 items: chances for pro-social participation (5), rewards for pro-social involvement (4), academic performance (2), commitment to school (6)	α = 0.70–0.76)	Strong construct validity
4	Community Environment	The community environment scale (CES)	M Gray and A Sanson [51]	Nine items related to a neighborhood environment	α = 0.84	Satisfactory construct validity
5	Subjective health literacy	HLS-CHILD-Q15	TM Bollweg, O Okan, P Pinheiro, J Bröder, D Bruland, AM Freţian, OM Domanska, S Jordan and U Bauer [52]	15-items, access, understand, and apply	α = 0.71 to 0.74	Significant
6.	Assessing health-promoting actions	Health-promoting actions questionnaire (HPAQ)	SN Walker, KR Sechrist and NJ Pender [53]	6 aspects; Self-actualization, health responsibility, nutrition, interpersonal support stress management, and exercise.	0.92 and	Significant

#### Qualitative data collection methods

To collect qualitative data, eight focus group discussions (FGDs) [54] will be held with students: four as a pre-test to get information regarding HL needs [40, 55], and four as a post-test to evaluate the outcome. Each FGD will last for an hour. Similarly, four key informant interviews (KIIs) with HPE teachers and school nurses from selected schools will be conducted before and after the intervention to get feedback on the intervention.

### Reliability, validity, and trustworthiness

First, HLS-Child-Q15 and other data collection tools will be translated by two professional translators using independent back-translation. After that, expert consultation will be received, and a Nepali version scale will be drafted. Then, the HLS-Child-Q15 will be pilot-tested qualitatively in nine adolescent students and modified based on the received feedback. Second, the pilot test will be conducted on 10 percent of total population for all standardized scales. They share the same features as the study area but are not incorporated into the study.

Participants’ opinions shall be properly documented with as much use of their own opinions as feasible for credibility. Themes will be explored and developed through author agreement. By contrasting and comparing data collected from students, HPE teachers, and school nurses for corroboration, we will triangulate qualitative data. Themes will be generated from the interview data by combining information from many sources (transcribe, memos, field reports, theory, and authors), and we’ll carry out member checks, requesting the participants to read the key themes and findings [54].

### Data collection procedure

The study will begin in the summer of 2022, at the beginning of the academic year. At two different points, data will be collected. Before and after the intervention is implemented in the study population, data will be collected simultaneously in both groups using the same way. Randomly, two schools will be selected from four to form the intervention group. The students from the remaining three schools will comprise the control group. After that, baseline data from both groups will be obtained. The characteristics of the intervention and control groups will then be matched based on the baseline data. Propensity Score Matching (PSM) will be used to obtain an unbiased estimation of the impact of the intervention and match the sociodemographic characteristics of both intervention groups [45]. Immediately after the intervention, the effect will be examined by post-test [56]. The self-reported questionnaire, and scales such as socio-demographic profile, HL knowledge, HLS-Child-Q15, HPA, and FGD for the students and KII for the teachers, and nurses will be employed to collect data related to the effectiveness of the intervention.

To collect quantitative data, students will use paper-and-pencil methods to complete self-reported scales. While conducting FGDs, a separate person will be employed as the note keeper and the principal researcher himself will moderate. Similarly, we will conduct KII to HPE teachers and school nurses about what they think is relevant regarding AHL. Based on the objectives and comprehensive model, semi-structured FGD and KII guidelines will be developed. Their information will be recorded with their consent.

### Data analysis procedure

For the formative assessment of HL needs, quantitative data will be analyzed by descriptive analysis. The software SPSS version 20.0 will be used to conduct all statistical analyses for quantitative data. Ritchie & Spencer’s (1994) five- steps framework analysis [57] will be applied to analyze the qualitative data from the pre-and post-tests. The verbatim recordings will be transcribed and ATLAS-TI software will be used for coding and categorization.

The summative evaluation methods will be used to compare pre-test and post-test results to determine if there has been any change in response. The Different in Different (DID) method will be applied to measure the impact of the program [58] which is also known as the double-difference method. Differences in the outcomes before and after intervention in both groups will be analyzed [45]. Mean, standard errors, mean differences, and a comparison of the results between the pretest and posttest will be examined.

### Ethical consideration

The Nepal Health Research Council (NHRC) approved this study protocol (NHRC, Ref. No. 2688). Students will be asked for written consent before participating in the study, and participation will be voluntary. Similarly, permission to record will be obtained, confidentiality and privacy will be ensured, and the COVID-19 protocol will be adhered to.

[59]. Approval will also be sought from the concerned authors for the adapting of the standardized tools.

## Discussion

The purpose of this research is to explore the design and impact of a school-based health literacy intervention for improving AHL and the intention to engage in health-promoting actions. To the extent of our knowledge, this is the first study to measure AHL needs and develop an intervention to promote AHL in schools in Nepal.

Previous studies reported that no intervention has explicitly aimed to improve HL[28]. The majority of existing school-based HL interventions are domain-specific without a holistic model [31]. It is emphasized that HL education may be increased by providing information, effective communication, media, organized education, and various methods in a range of settings [60]. HL education aims to increase knowledge, to promote health-promoting attitudes and beliefs (KAB framework) [31]. School adolescents have a low HL level and health-promoting activities. HL is a concept that focuses on education and training to improve the HL of individuals and populations, both individually and collectively [61]. According to our understanding, there were few interventional studies on HL and health-promoting actions in adolescent students. These findings provide evidence that an HL intervention may contribute to promoting HL and health-promoting actions in adolescents.

The intervention’s strengths include a school-based health literacy intervention and execution in the school context. Furthermore, the Ministry of Health and Education, as well as other related sectors, may use the findings of this study to support policy and planning to enhance health literacy among school-aged adolescents. The intervention’s components will be matched to the school’s HPE curriculum and the participants’ needs. This study incorporates a comprehensive model of HL to guide, embed, and respond to the participants’ health literacy needs and interventions. This research will provide preliminary evidence that school-aged adolescents who received an HL intervention increased their HL knowledge, competencies, and intention to engage in health-promoting actions.

The main limitation of this study will be that it is not properly randomized and only took place in the Surkhet district of Nepal. Because participants were asked survey questions, we could not rule out the possibility of measurement errors. This study does not offer a multi-level approach to changing environmental and societal factors. More research is needed to develop an effective intervention to address these societal and environmental factors.

## Conclusion

It would be very useful to study HL among young people in schools. A study will be conducted to determine the health literacy needs of adolescents. School-based health literacy interventions may influence adolescents’ health literacy and health-promoting behaviors.

## Data Availability

Not applicable as this article does not present data.

## Abbreviations

AHL: Adolescent Health Literacy
FGD: Focus Group Discussion
HL: Health Literacy
IMHL: Integrated Model of Health Literacy
KII: Key Informant Interview
HPE: Health and Physical Education
KAB: Knowledge, Attitude and Behaviour
NHRC: The Nepal Health Research Council
DID: Different in Different
HLS-Child-Q15: Health Literacy Scale- Child Questions 15
HPA: Health promoting Actions
PSM: Propensity Score Matching
SSS: Standard Sample Size
HLEI: Health Literacy educational intervention
HPS: Health Promoting School
